# Two Bee-Pollinated Plant Species Show Higher Seed Production when Grown in Gardens Compared to Arable Farmland

**DOI:** 10.1371/journal.pone.0011753

**Published:** 2010-07-23

**Authors:** John Cussans, David Goulson, Roy Sanderson, Louis Goffe, Ben Darvill, Juliet L. Osborne

**Affiliations:** 1 Plant and Invertebrate Ecology Department, Rothamsted Research, Harpenden, United Kingdom; 2 School of Biological and Environmental Sciences, University of Stirling, Stirling, United Kingdom; 3 School of Biology, Newcastle University, Newcastle upon Tyne, United Kingdom; Trinity College Dublin, Ireland

## Abstract

**Background:**

Insect pollinator abundance, in particular that of bees, has been shown to be high where there is a super-abundance of floral resources; for example in association with mass-flowering crops and also in gardens where flowering plants are often densely planted. Since land management affects pollinator numbers, it is also likely to affect the resultant pollination of plants growing in these habitats. We hypothesised that the seed or fruit set of two plant species, typically pollinated by bumblebees and/or honeybees might respond in one of two ways: 1) pollination success could be reduced when growing in a floriferous environment, via competition for pollinators, or 2) pollination success could be enhanced because of increased pollinator abundance in the vicinity.

**Methodology/Principal Findings:**

We compared the pollination success of experimental plants of *Glechoma hederacea* L. and *Lotus corniculatus* L. growing in gardens and arable farmland. On the farms, the plants were placed either next to a mass-flowering crop (oilseed rape, *Brassica napus* L. or field beans, *Vicia faba* L.) or next to a cereal crop (wheat, *Triticum spp.*). Seed set of *G. hederacea* and fruit set of *L. corniculatus* were significantly higher in gardens compared to arable farmland. There was no significant difference in pollination success of *G. hederacea* when grown next to different crops, but for *L. corniculatus*, fruit set was higher in the plants growing next to oilseed rape when the crop was in flower.

**Conclusions/Significance:**

The results show that pollination services can limit fruit set of wild plants in arable farmland, but there is some evidence that the presence of a flowering crop can facilitate their pollination (depending on species and season). We have also demonstrated that gardens are not only beneficial to pollinators, but also to the process of pollination.

## Introduction

The anthropogenic introduction of large quantities of flowering plants has occurred both in arable and urban habitats. In urban gardens high densities of flowering plants are cultivated for their amenity value, while in agriculture the cultivation of mass-flowering crops such as oilseed rape (*Brassica napus* L.) and field beans (*Vicia faba* L.) in the UK has been arguably the most dramatic change to the floral landscape for centuries. Oilseed rape crops began to be cultivated on a large commercial scale in the mid-1970's in the UK and are now grown on an unprecedented scale. In 2009 oilseed rape was cultivated on approximately 15% of UK arable land (∼600 000ha [1]), and it has become a familiar part of our spring landscape, yet the potential ecological impacts of this change in agricultural practice are only just starting to be recognised [2], [3], [4], [5]. It is possible that, during the flowering period, the nectar and pollen provided by these crops greatly exceeds that provided by all other flowers combined in arable landscapes. A similar situation exists in urban gardens where floral resources provided by cultivated plants are plentiful over large areas. In the case of garden plants though, there tends to be nectar and pollen available from different species through most of the year [6] whereas the mass-flowering crops only provide resources from one species over a relatively short period of a few weeks.

For mass-flowering crops, there is sparse evidence of the impact that this brief glut of floral resources has on the seed or fruit set of flowers that share pollinators with the crops. Do they impact negatively on levels of seed set in wild flowers in neighbouring hedgerows through competition for pollinators, or do they have a positive impact (facilitation) through attracting more pollinators to the area and thus boosting pollination and subsequent seed production? Diekotter et al [2] found no effect of the proportion of landscape covered with oilseed rape on seed set in red clover (*Trifolium pratense* L.); although they did detect a positive effect of the amount of semi-natural vegetation in the area. In contrast, our study examines the local effect of a neighbouring crop, and utilises plant species that share pollinators with the crops. There has been growing interest in the possible effects of co-flowering plant species on pollen limitation [7], [8], [9], particularly in relation to invasive alien species of flowering plant affecting native plant species. Evidence of effects is variable, with some species positively affected, others negatively [10], [11], [12], [13]. The direction of a response in seed or fruit production is likely to vary depending on the species of pollinators and the species of wild flowers.

In agricultural settings there is some evidence that the species of the crop sown in the field, or the management thereof, can influence the abundance of bumblebees and other pollinators in the field margin in the short term as a result of re-distribution of bees [3], [5], [14] and possibly in the longer term as a result of increase colony density and growth [4], [15].

If there are effects on plant pollination (positive or negative) then they may be temporally localised if they are a result of changed pollinator behaviour, for example if their choice of forage is changed and they are attracted into the area in large numbers. Alternatively the effects may be spread out over the season if the copious floral resources (in gardens or farmland) are driving changes in the insect pollinators' population dynamics. The variety, abundance and continuity of floral resources provided in gardens is thought to positively affect bumblebee populations [6], [16], [17], [18]. Against a backdrop of evidence that urbanization generally leads to species loss and reduction in biodiversity [19], [20] there is increasing evidence pointing to the potential for urban areas to act as a refuge for certain bumblebee species. Several studies have found bee abundance and diversity to be high in urban and suburban areas [21], [22] although it depends on the degree of urbanisation [23], [24] and Osborne et al [25] found there was a higher density of bumblebee nests in gardens compared to largely agricultural countryside. In addition Fetridge, Ascher & Langellotto [26] found the bee (Apoidea) fauna of suburban gardens closely resembled that found in nearby natural forest. Goulson et al [27] found that the survival of bumblebee nests from May to August was positively associated with the area of gardens in the vicinity of the colony. This relatively positive picture for social bees may be at odds with the global picture of urbanization but, for these species at least, urban areas can provide an important resource for population survival. This positive impact of gardening on bumblebee populations may have a knock-on effect on pollination levels of plants growing in the surrounding environment.

Although studies comparing urban and agricultural settings have assessed bee abundance, as have studies comparing the field margins of mass flowering crops with other crops, to our knowledge no studies have reported on the relative seed production of insect-pollinated wild plants growing in these different settings. We carried out a replicated experiment to determine whether mass-flowering crops and the floral resources in gardens have a competitive or facilitatory effect on pollination and seed-set of *Glechoma hederacea* L. (ground ivy) and *Lotus corniculatus* L. (birdsfoot trefoil). These species were selected because they are entirely or largely self-incompatible, relying on insect pollination, primarily by social bees [28], [29], [30]. They vary in morphology and phenology and occur naturally in field margins and hedgerows in the study area.

We tested the null hypothesis that pollination, and resultant seed and fruit set of *G.hederacea* and *L.corniculatus* were not different when the plants were growing in contrasting locations. If the null hypothesis is rejected, we predict one of the two following outcomes for the wild flowers: 1) the presence of a mass flowering crop in the near vicinity or placement in a suburban garden has a facilitatory effect on pollination, resulting in increased fruit or seed-set; or 2) the presence of a flowering crop or placement in a suburban garden reduces pollination and consequent fruit or seed-set, because of competition and increased pollen limitation [9].

We tested these predictions using oilseed rape and field beans. Seed yield in both crops is increased by insect pollination (although self pollination also occurs, and wind pollination in oilseed rape), and when pollinated by insects the two crops have contrasting pollinator guilds. Oilseed rape is pollinated by short-tongued pollinating insects (including honeybees) whilst field beans are pollinated by long-tongued bumblebees (although the flowers are frequently robbed by short-tongued bees). The control treatment was winter wheat which is not visited by bees. In addition a comparison was made with suburban domestic gardens. We also quantified relative abundance of flowers and bees in the vicinity, because our hypotheses assume that changes in the relative abundance of pollinators would be the likely mechanism for increased or decreased seed and fruit set in the experimental plants. The results will increase our ability to predict the impact of agricultural practices and urbanisation on populations of wild plants in the landscape.

## Materials and Methods

*G.hederacea* and *L.corniculatus* plants were bought as small plugs and reared in a glasshouse to ensure they were of similar age, provenance and growth stage. The plants were transferred into large, 25cm pots which were placed in 80 litre tubs of sand (60cm in diameter). Plants in natural populations are highly variable, depending on the conditions in which they grow, so we used pot-grown experimental plants to ensure that, as far as possible, resources were controlled to prevent differences in plant growth and development between treatments and sites.

**Figure 1:**
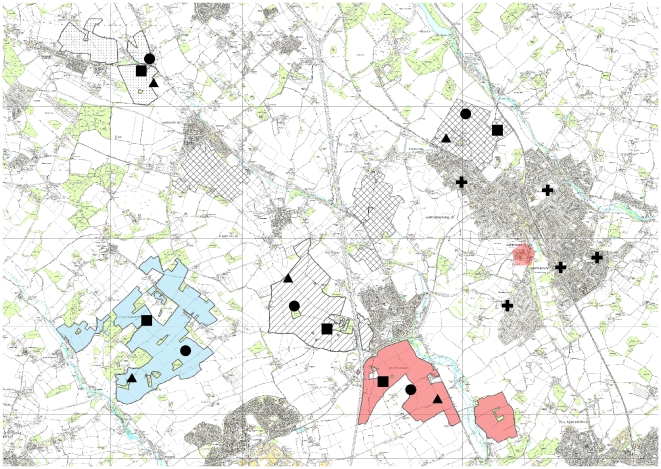
Map of experimental sites. The extent of each farm hosting the field margin sites is shown in contrasting shading. Field bean sites are indicated with circle symbols, oilseed rape sites with triangles and wheat sites with squares. The garden sites are shown with crosses. The area of the map is entirely located within Ordnance Survey square containing Rothamsted Research (TL 13415 13598). Sites on different farms are a minimum of 1750 m apart. Sites next to contrasting crops on the same farm fit within a circle of radius 1000 m. The garden treatment sites are all within the Harpenden town conurbation. © Crown Copyright/database right 2010. An Ordnance Survey/EDINA supplied service.

The experimental sites were on 15 field margins on commercial farms within 10 km of Rothamsted Research station, Harpenden, Hertfordshire, UK (Ordnance Survey coordinates TL 13415 13598) and five domestic garden sites in the urban area of Harpenden (Fig 1). In April four tubs, each containing one *G. hederacea* plant and one *L. corniculatus* plant, were placed in a group (subsequently referred to as a patch) at each of the 20 sites. For each species, the number of flowers in these patches gave densities similar to the sparse and small natural patches found in the field margins (see results).

The field margins were on five farms (considered as blocks in the analysis) which were at least 1750 m apart and there were three crop treatments on each farm (Fig 1). So within each block there was a patch of tubs on a margin adjacent to winter oilseed rape, one adjacent to winter field beans and one adjacent to wheat (the control). These margins were located within a circle of 1000 m on each farm. We consider the blocks to have insect pollinators from different colonies or populations as most individuals are likely to fly less than the separation distance of 1750 m [31], [32]; but within each farm, the same individuals are likely to be choosing between treatments. It was not feasible to spread the sites more widely. The field margins were each 4–6 m wide, sown with grass or naturally regenerated with a mixture of grass/herbaceous species and adjacent to a hedgerow. The tubs were placed adjacent to the hedge to prevent shading from the different crop treatments.

At each of the five domestic garden sites (Fig 1), a patch of four tubs was placed adjacent to a border or boundary. It is difficult to ensure comparability between the farmland and garden sites because of the different structures of these habitats. However, we aimed to make them as comparable as possible by using the most “linear” features in each garden, for example a herbaceous or perennial border next to a lawn, or a boundary hedge. At all 20 sites (in both farmland and gardens), the tubs were positioned adjacent to south-facing boundaries to reduce the effects of differential shading or shelter between sites.

When the plants were placed at the sites, they were all of similar size and growth stage; and the soil and space available to the plants were matched. During the course of the experiment a watering system was set up so that all the tubs were maintained at the same soil moisture. These steps were taken to ensure that the plants' access to nutrients, water and light were controlled and comparable among treatments and sites. Records of flower abundance, bee visitor abundance and seed set were taken during each of four observation periods from April to August. Each period was about four weeks long to fit in all the observations required (start dates: 18 April, 25 May, 25 June, 27 July)

### Test for self-incompatibility

In a separate experiment seed set in plants grown in insect-proof cages versus open-pollinated plants was compared to confirm that the plant species used were at least partially self-infertile (this can vary between races and populations) and require insects to mediate pollen transfer. Twelve plants each of *L. corniculatus* and *G. hederacea* were grown. At the point at which the plants began to flower, six plants of each were transferred into an insect-proof cage. On each plant, 15 flowers were individually marked with coloured tape (Scotch® 35 Colour Coding PVC Electrical Insulation) and the seed or fruit set for each flower was assessed as described below.

### Local flower abundance

During each of the four observation periods an assessment was made of the flower density of a) the sown wild plants in tubs, b) the crop and c) other flowering plants in the margins or garden border next to the tubs. The number of flowers of each plant species present in a 200 m length of each field margin were counted (Table S1). In each garden, a transect counting flowers of each plant species was also walked during each period. The transect incorporated the garden boundary or border next to the tubs and utilised other linear features in the garden (e.g. around the perimeter). In some cases it was not possible to walk a 200 m length (we only had access to individual gardens) so the resulting data are expressed per 200 m to make comparisons with the field margins. Every plant species encountered during the margin transects was assigned a score for the likely usage by bumblebees and honeybees: 0 = not used as forage; 1 = used as forage (Table S1). These scores were assigned using the methods of Osborne et al [32] who examined records in comparative forage studies and reviews and using the combined observational experience of the authors. Visitation records were verified using Knuth [30], [33], [34]. A score of zero represented an absence of positive records of visitation by bees in any of the above references. Species given a score of 1 were included in the list of “bee forage plant species” and used in the analysis. It was not possible to record attractiveness or reward levels in further detail in this experiment.

### Local flower visitor abundance

To assess the local abundance of potential pollinators, flower visitors were surveyed by observing the number and species of all flower visits taking place along the same length of field margin or garden boundary that was used to assess local flower abundance using a standard walk, between 10.00 h and 17.30 h in standard weather conditions (temperature above 13°C with at least 60% clear sky or above 17°C in any sky conditions apart from heavy rain; Beaufort wind speed of less than 5) [3]. One transect was performed during each observation period. Counts of the number of insects visiting the experimental patches of plants were taken during 4×10 minute sessions spent watching each patch during each observation period during standard weather conditions (above), but the numbers were too low for analysis.

### Seed and fruit production

The number of flowers produced on the plants in tubs during each of the four observation periods was determined by marking the stems with coloured tape (Scotch® 35 Colour Coding PVC Electrical Insulation) at the beginning and end of the period, and counting the number of flowers in between the coloured tape marks. Seed heads were gathered from these marked stems before seed shed in order to assess seed production. When they were gathered, seed heads that showed signs of herbivory or contained larvae were not included in the analyses. For *G. hederacea* an average of 352.5 (±17.3) flowers per patch were collected in each of the first two sampling periods (n = 40). A count was made of the number of seeds formed in each flower (with four ovules), including flowers which produce no seeds (from here on described as ‘seed-set’). The seeds mature at different rates both within and between the plant species. *G.hederacea* stems were sampled 1–2 weeks after the flowers were counted, when the “youngest” seeds near the tops of the stems were swollen and green. The “older” seeds further down the stem were mature and some had already been shed, but it was possible to score them from the scars left at the flower base. There were not enough *G. hederacea* flowers present during the third and fourth observation period to collate seed-set data. *L.corniculatus* ripe fruits were collected approximately four weeks after the flowers were marked and counted. A mean of 96.9 (±3.9) flowers were sampled from the four plants in each patch during each time period (n = 80). The proportion of these flowers producing fruits was counted (from here on described as ‘fruit-set’). A repeated measure ANOVA showed that the number of flowers sampled per patch was not significantly different between treatments or observation periods for either plant species.

### Statistical analysis

Repeated measures ANOVAs were used to examine the effect of treatment and observation period on the abundance of bee forage flowers (Table S1) in the margins, social bee abundance along the margins and experimental plant seed or fruit production. The bee abundance data were transformed to log_10_ (bees+1) because the data were highly skewed and the transformation ensured the data fitted assumptions of normality more closely. An additional variable was derived weighting bee abundance by forage availability ( = log_10_ (bees+1)/no. bee forage flowers) and a repeated measures ANOVA was also performed on this. With the experimental design described, two statistical comparisons were possible: 1) the comparison between urban gardens and arable field margins, and 2) the comparison between the three different arable crops. It should be noted that because of the spatial design of the experiment (farms as blocks, and gardens in a different area) it was not possible to make statistical comparisons between individual arable crop margins and urban gardens. The repeated measures analyses were also used to test if there were interactions between these treatments effects and the observation periods. For *G. hederacea*, an average value of seed set per flower was used for each patch (1 patch×4 treatments×5 sites×2 time observation periods). For *L.corniculatus*, an average proportion of flowers setting fruit per plant was used, and there were 4 plants per patch (4 plants per patch×4 treatments×5 sites×4 observation periods). Since the number of sampled flowers was so high, the data (although proportional) were approximately normally distributed and did not require transformation.

The experiment was structured for the above analyses, and it was not statistically appropriate to include flowers as a co-variate in the bee analysis; or to include bees as a covariate in the seed or fruit analyses. Instead, and in order to explore the observed patterns more fully, three simple regressions were performed (post-hoc). For the margin/border data, the relationship (at the site level) between bee numbers (log_10_ (bees+1)) and margin bee forage flowers was examined using linear regression. For the pollination data, linear regressions (at the site level) were performed for a) *G.hederecea* seed set and margin bee abundance; b) *L.corniculatus* fruit set and margin bee abundance.

## Results

### Test for self-incompatibility

For *G. hederacea* the number of seeds produced per flower was significantly higher (Mann-Whitney U test: P = 0.02, n = 12) in the open-pollinated plants (mean = 3.53±0.25) compared to the caged plants (mean = 0.05±0.05). The proportion of *L. corniculatus* flowers producing fruits was significantly higher (Mann-Whitney U test: P = 0.02, n = 12) for open-pollinated plants (mean = 0.88±0.05) than for caged plants (0.13±0.04). This effectively demonstrates that both of the populations of plants used in this experiment benefited significantly from insect visits to set fruit and seed.

### Local flower abundance

The oilseed rape crop flowering coincided with the first observation period (April–May), and the first part of the second period (May–June). The field bean crop flowering coincided with the second observation period (May–June). From the field margin and garden border transects, *G.hederacea* was observed growing naturally at eight of the 20 sites (1 garden and 7 field margins). The average density of the species (where it occurred) was 51 flowers per 200 m of garden border and 48 flowers per 200 m of arable field margin. For *L.corniculatus* the number of sites where plants were observed in the vicinity of the experimental tubs was four (1 garden site and 3 field margins). The average density of the species (where it occurred) was 388 flowers per 200 m of garden border and 278 per 200m of field margin transect.

**Figure 2:**
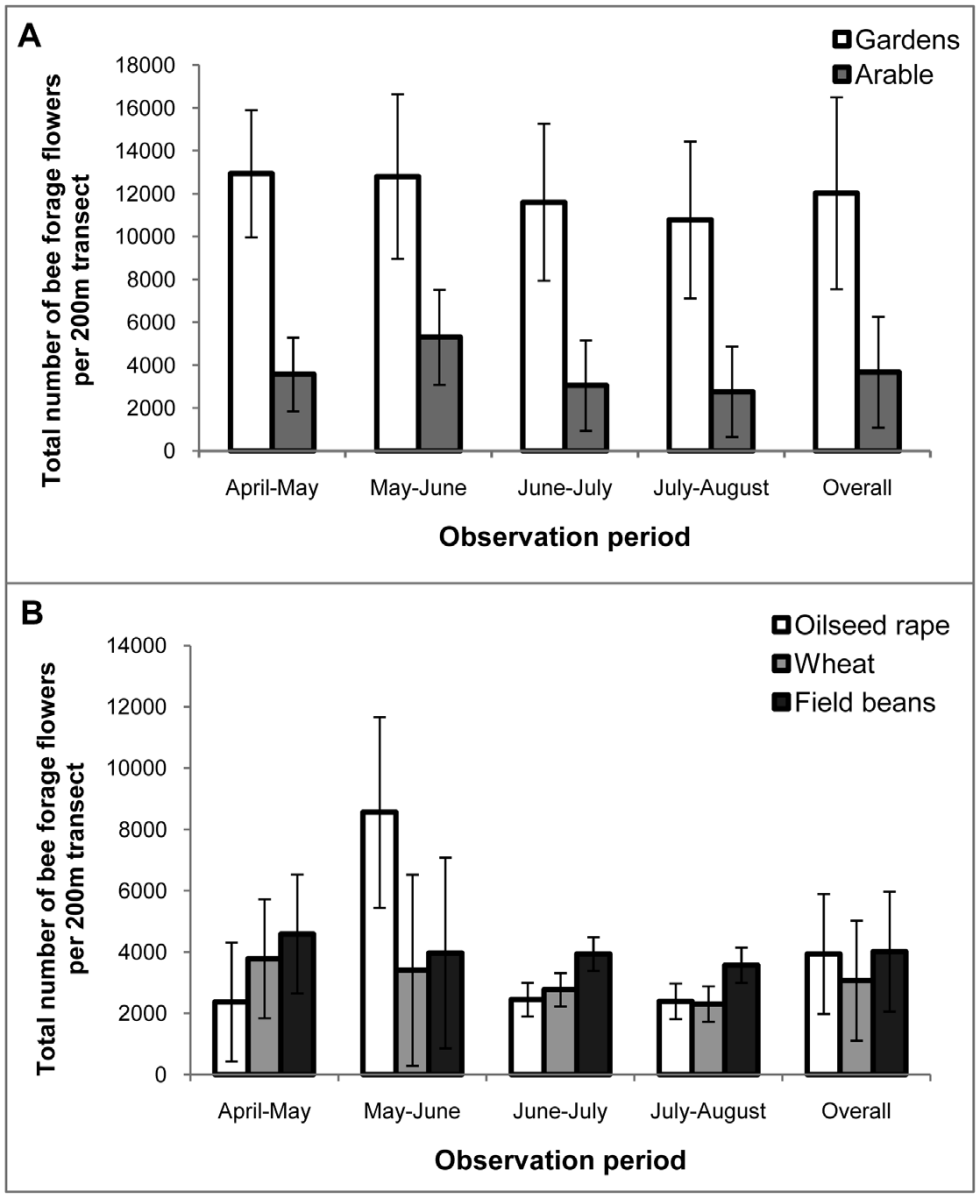
Bee forage flower density in field margins and garden borders. Average number of flowers (±s.e.m.) of bee forage flower species in field margins and garden borders (expressed per 200 m of transect length) for each observation period. **A** Comparison between garden borders and arable field margins; **B** Comparison of the abundance of flowers in the margins of three different arable crops.

For bee forage plant species in the 200 m margin and border transects (Table S1), there were significantly more flowers in garden borders than in field margins (of oilseed rape, wheat and bean crops) over all observation periods (Fig 2A; F_1,8_ = 5.39; P = 0.049;). There were no significant differences between the average number of bee forage flowers per 200 m observed in the margins of the different arable crops (Fig 2B) and no significant interaction between the observation period and the treatment effects.

### Local flower visitor abundance

The frequency of insect visits to the experimental plants was low such that the data were too few to analyse statistically. Qualitatively, *G. hederacea* received most visits (total for observations given in brackets) from *Bombus hortorum* (17) and *Bombus pascuorum* (14), with some visits from *Bombus terrestris/lucorum* (7; not separated taxonomically) and *Bombus lapidarius* (2). *L. corniculatus* received most visits from *B. pascuorum* (25) with a few visits from unidentified solitary bees (5), *B. lapidarius* (3) and *B. hortorum* (1).

**Figure 3:**
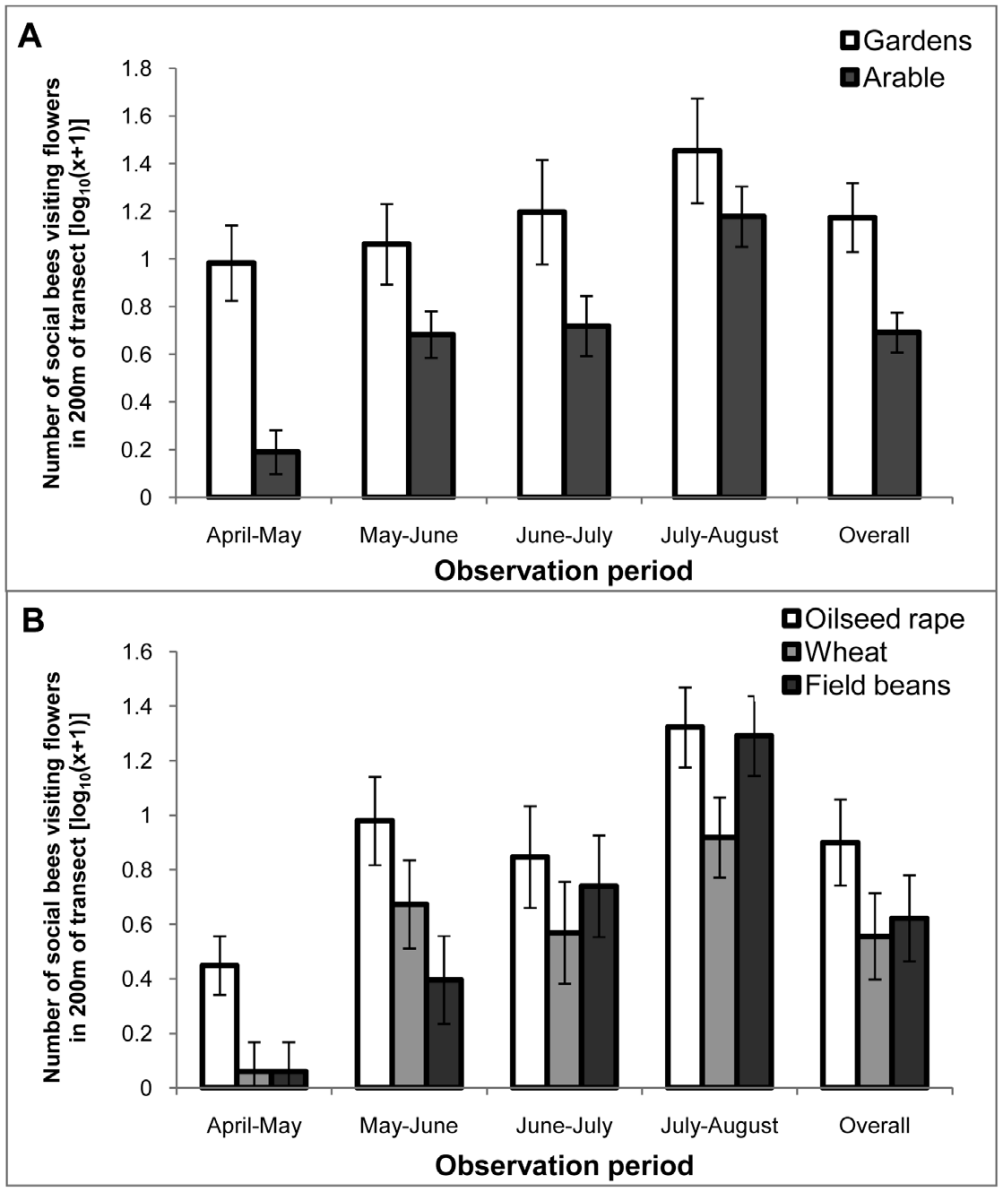
Density of social bees visiting flowers in field margins and garden borders. Mean number of social bees (±s.e.m.) observed visiting flowers in arable field margins or garden border (expressed per 200 m of transect length) for each observation period. **A** Comparison between garden borders and arable field margins; **B** Comparison of the number of bees visiting flowers in arable field margins of three different crop species.

Table 1 shows the number of flower visitors belonging to different insect groups observed in the field margin and garden border transects. The number of individuals of each species was low, so they have been combined into bumblebees, honeybees, solitary bees and other visitors (a group dominated by small flies). Most visitors were social bees (bumblebees and honeybees) and since these are considered the most likely pollinators, and were observed on the experimental plants, we focussed our analysis on this group. Significantly more social bees were observed visiting flowers in the garden borders than in the arable field margins (Fig 3A; F_1,8_ = 8.33; P = 0.02). There were no significant differences between the average number of social bees foraging per 200 m of margins of the different arable crops (Fig 3B) and no significant interaction between the observation period and the treatment effects.

**Table 1:** Types of insect flower visitors observed on field margins and garden borders.

	Garden border	Arable field margin
Bumblebees	13.20±2.58	7.98±1.57
Honey bees	5.63±1.18	1.13±0.34
Solitary bees	0.95±0.70	1.53±0.41
Other flower visitors	7.6±5.21	18.3±3.01

The average number of insects observed visiting flowers in transects along garden borders and arable field margins (expressed as per 200 m transect length). Values given are means of all four observation periods (±s.e.m.). In all cases n = 20 for garden border, and n = 60 for arable field margins.

The number of social bees (log_10_(x+1)) per margin or border transect was positively and significantly correlated with number of bee forage flowers per transect (n = 20; R^2^ = 0.64, P<0.001). When a repeated measures ANOVA was performed on the ratio of bees to flowers (log_10_ (bees+1)/no. bee forage flowers), then there were no significant differences between gardens and arable field margins (F_1,8_ = 2.83; P = 0.131), suggesting that the higher relative abundance of bees at the garden sites was partly due to the increased number of bee forage flowers available.

### Seed and fruit production

**Figure 4:**
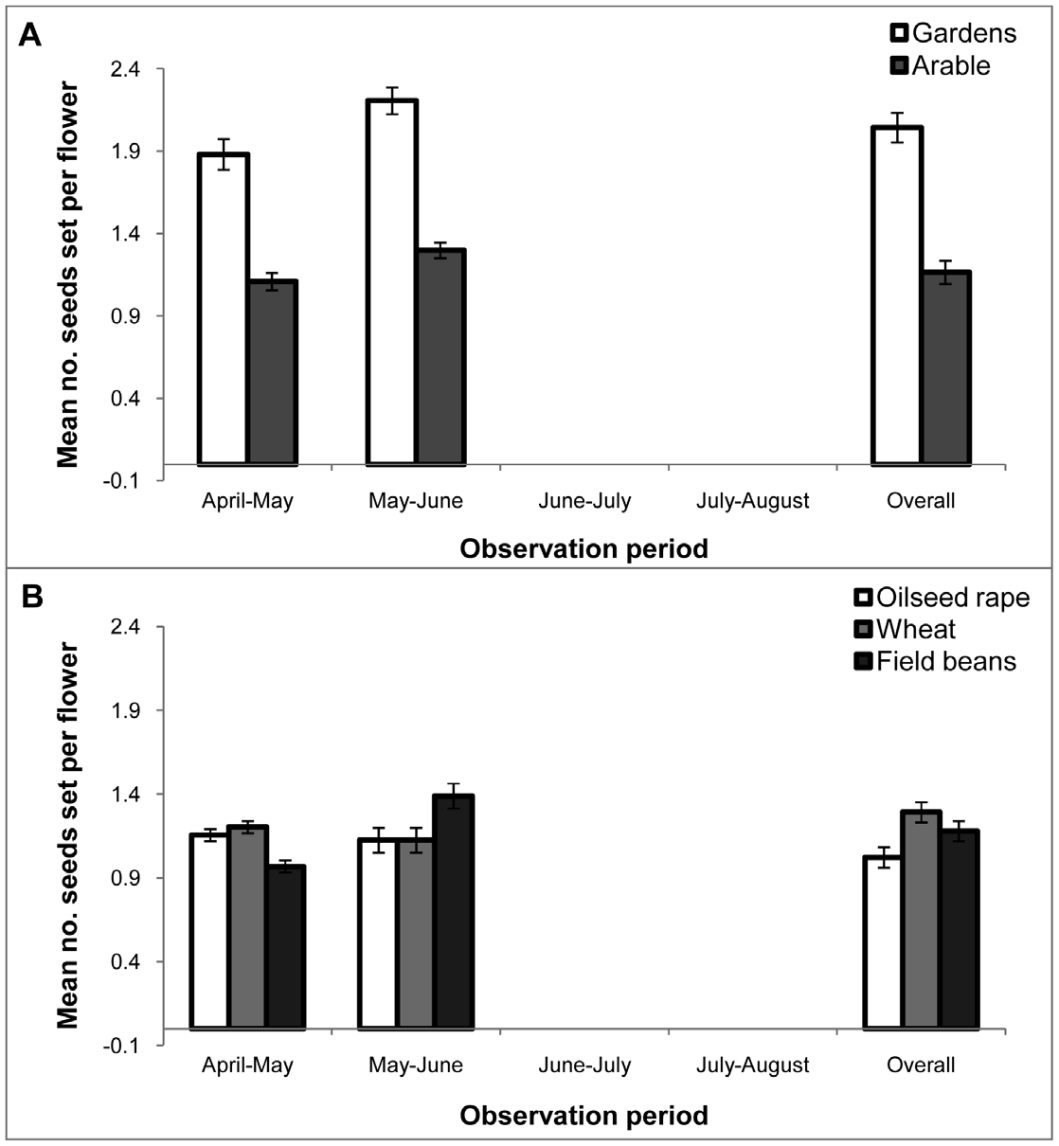
Seed set in *G.hederacea* (ground ivy) plants growing in different habitats. Average number of seeds set per flower (±s.e.m.) in ground ivy (*G. hederacea*) which only flowered during the first two observation periods. **A** Comparison between plants in gardens and those in arable habitats; **B** Comparison of seed-set for plants grown next to three different arable crops.

During the first two observation periods (which coincided with oilseed rape and field bean flowering times respectively) there was significantly higher *G. hederacea* seed set in gardens than in the arable field margin settings (Fig 4A; F_1,8_ = 7.07; P = 0.029). Neither the observation period nor the placement next to different arable crops had a significant effect on *G. hederacea* seed set (Fig 4B).

**Figure 5:**
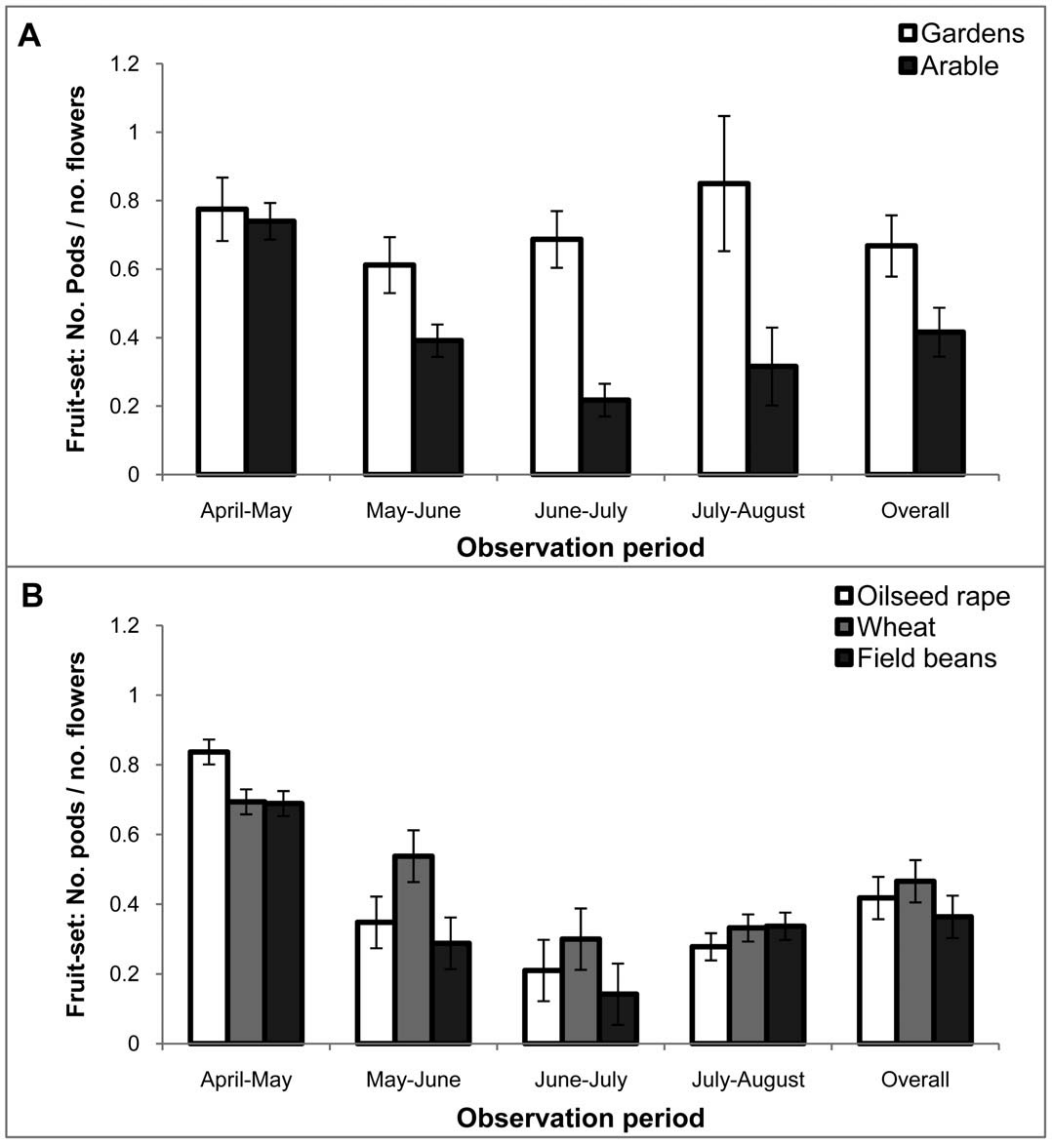
Fruit set in *L.corniculatus* (birdsfoot trefoil) plants growing in different habitats. Average proportion of pods setting fruit (±s.e.m.) in bird's foot trefoil (*L. corniculatus*) in different observation periods. **A** Comparison between plants in gardens and those in arable habitats; **B** Comparison of fruit-set for plants grown next to three different arable crops.

For *L. corniculatus*, fruit-set was also significantly different between the gardens and the arable field margin setting (Fig 5A; F_1,8_ = 7.69; P = 0.02). For this species there was also a significant interaction between the treatment (garden versus arable) and the observation period (F_3, 197_ = 12.50; P<0.001). Fruit-set was consistently high over the season for plants placed in gardens, but was lower in later observation periods for plants placed in the arable margins. The contrast between garden and arable locations was highest at the 4^th^ sample date (Fig 5A). For the plants placed next to field margins, there was no significant difference in fruit set between crop treatments, but there was a significant interaction between observation period and crop treatment (Fig 5B; F_6, 197_ = 2.94; P = 0.014). The strongest pattern was seen for the *L. corniculatus* plants situated next to oilseed rape fields where fruit set was highest in April–May when the oilseed rape was in flower, and then lower in the following observation periods (Fig 5B).

Linear regressions showed that *G.hederacea* seed set was significantly positively correlated with the number of bees observed visiting flowers in the margins and borders (n = 20; R^2^ = 0.24, P = 0.017) and *L.corniculatus* fruit set was also significantly positively correlated with bees in the margins (n = 20; R^2^ = 0.15, P = 0.048).

## Discussion

For two plant species, *G. hederacea* and *L. corniculatus* for which seed set is significantly enhanced by insect pollination, measurements of seed and fruit set (respectively) showed there were significantly higher levels of pollination in plants growing in tubs in gardens, compared to those growing in tubs in arable field margins in Hertfordshire (Fig 4; Fig 5). The pollination in gardens was consistently higher throughout the season, as was the density of other flowers in the locale (Fig 2) and the number of pollinating insects visiting these other flowers (Fig 3).

This effect on seed and fruit set could be a result of differing patterns of insect pollination: including visit quantity or quality. Unfortunately the sampling effort on the tubs did not give enough data on insect visitation rate to experimental plants to allow correlations to be made. Interpretation of the patterns is therefore made with caution using the surrogate measure of the abundance of social bees foraging in the adjacent margin or border, and the abundance of co-flowering bee forage plants in the margin (and the presence or absence of a flowering crop). There were more co-flowering forage plants in the gardens than in the arable margins (Fig 2), and there were relatively more bees foraging in the garden borders than in the margins (Fig 3). These figures, combined with the seed and fruit set data suggest that there is a facilitatory effect of other co-flowering plants within the gardens, providing a good “pollination environment” for the experimental plant species. The co-flowering species attracted foraging bees into the vicinity in proportion to the floral abundance (there was a high correlation between flower abundance and bee abundance). When the data for social bee abundance in margins were expressed as the number of bees per flower, there was no significant difference between treatments. Thus in this experiment, the number of bees per 200 m (Fig 3A) which did vary significantly between treatments, was the more suitable variable to be correlated with seed and fruit set in both species. The significant positive correlations suggest that some of the differences in seed and fruit set can be explained by local pollinator abundance per unit area (rather than the number of bees per flower).

It is possible that other differences in abiotic and biotic conditions between field margins and garden borders also contribute to differences in seed and fruit set, but the experiment was designed to keep abiotic conditions (e.g. microclimate, shading and resources) as constant as possible. Herbivory and seed predation could also be important factors in overall reproduction of the plants but were not responsible for the observed effects because flowers that showed herbivory damage, or contained larvae, were removed from the samples before the seed and fruit counts were done.

It is also likely that the characteristics of urban areas that lead to the higher abundances of bees reported here and by others [6], [22] go beyond the availability of forage. One factor (highlighted in [6]) is that gardens and parks in urban areas provide a robust and diverse supply of forage for pollinators throughout the year (Fig 2A). The availability of safe sites for nests is also a key feature of urban areas [25] so that the overall population levels are higher than in an arable setting (although see [23]). It is not possible from our results to say what the causal mechanism is; and it could be a combination of these factors.

In arable farmland, we found seed and fruit set levels in both species were lower than in the gardens, suggesting some degree of pollen limitation at our study sites. There was also some evidence that flowering oilseed rape had a facilitatory effect on *L. corniculatus* fruit set in the first observation period (since there was a significant interaction between crop and observation period), an effect that was not sustained after the flowering of the crop. This is suggestive of the hypothesis that a mass-flowering crop attracts pollinators into the area to the benefit of other plants, and the lower seed set later in the season is suggests that this local boost is not maintained through the season although more highly resolved data on bee densities and visitation patterns on the crops would be required to confirm or disprove these suggestions. Bumblebees fly long distances to find forage [35], [36] and, even if they have a successful nest in the margin next to a crop, they may not stay in the vicinity to search for small patches of forage (such as our experimental plants) if there are larger, more profitable patches at a further distance [37].

Our results are specific to two species of experimental plant, both chosen as plants favoured by bumblebees but with differing phenology and floral attributes. It is interesting to note that the only significant interaction with crop type was a positive one between oilseed rape and *L.corniculatus*. Both have yellow flowers and, although they differ considerably in morphology and olfactory cues, they are both frequently visited by short-tongued bumblebees and thus “share” a pollinator guild. *G. hederacea* may be more dependent on bumblebees with longer tongues (it was most visited by *B.hortorum* and *B.pascuorum* in this experiment) and so shares a pollinator guild with field beans, although the flowers are markedly different in colour and no effect on seed set was seen in combination with this crop. This is similar to the results of Diekotter et al [2] who studied *T. pratense*, another species pollinated by long-tongued bumblebees (although this flowers much later than the crops).

We found no evidence of competition either between mass-flowering crops and experimental plants in the field margins for pollinators; or between garden plants and experimental plants for pollinators. If the interactions were competitive we would, in theory, have expected the number of bees per flower in the margins to be lower in the treatments where there were most bee forage flowers available (e.g. in gardens and when the oilseed rape was flowering) and consequently the more abundant flowers would have to compete for pollinator visits, but this was not observed. Although we have studied different habitats, our results support those of Hegland et al [38] who showed, for bumblebee visitation rates of grassland plant species, positive plant intra-specific and inter-specific interactions were far more frequent than negative ones. In summary, there is evidence that plants growing in small patches, in the vicinity of large quantities of anthropogenically introduced flowers, may have increased seed or fruit set but this will depend on the floral phenology and attributes. In particular, gardens in Hertfordshire seem to be a beneficial environment for pollination by bees, compared to the arable farmland surrounding the town, irrespective of crops growing in the fields.

## Supporting Information

Table S1 List of plants recorded in field margins and garden borders. Plants are listed according to whether they were recorded only in the field margins (F), in both field margins and garden borders (F&G) or in only the garden borders (G). In the garden borders it was not possible to identify all the plants to species so these species are grouped into genera. If a species is likely to be visited by bees then it is given a score of 1 (see explanation of in the methods), and these “bee forage plant species” species were used to estimate the flowers available to bees per 200 m transect in the analysis. If bees were actually observed visiting the plant species during the transects, then a Y appears in the 4th column. The final column indicates the number of experimental sites at which the species was recorded.
(0.18 MB DOC)
