# Supplementary material for: Two Bee-Pollinated Plant Species Show Higher Seed Production when Grown in Gardens Compared to Arable Farmland
Source: PLoS One. 2010 Jul 23;5(7):e11753. doi: 10.1371/journal.pone.0011753 (PMC2909262; doi:10.1371/journal.pone.0011753)
Supplement: Table S1 — List of plants recorded in field margins and garden borders. Plants are listed according to whether they were recorded only in the field margins (F), in both field margins and garden borders (F&G) or in only the garden borders (G). In the garden borders it was not possible to identify all the plants to species so these species are grouped into genera. If a species is likely to be visited by bees then it is given a score of 1 (see explanation of in the methods), and these “bee forage plant species” species were used to estimate the flowers available to bees per 200 m transect in the analysis. If bees were actually observed visiting the plant species during the transects, then a Y appears in the 4th column. The final column indicates the number of experimental sites at which the species was recorded. (0.18 MB DOC) [file pone.0011753.s001.doc]

**Table S1. List of plants recorded in field margins and garden borders.**

| **Plant Species** | **Recorded in field and/or gardens** | **Likely usage by bees** | **Observed visitation by bees** | **No. sites where recorded** |
| --- | --- | --- | --- | --- |
| *Achillea millefolium* (yarrow) | F | 0 |  | 3 |
| *Aethusa cynapium* (fools parsley) | F | 0 |  | 5 |
| *Agrimony eupatoria* (common agrimony) | F | 1 |  | 3 |
| *Alliaria petiolata* (Jack-by-the-hedge) | F | 0 |  | 4 |
| *Anagallis arvensis* (scarlet pimpernel) | F | 0 |  | 3 |
| *Arabidopsis thaliana* (thrale cress) | F | 0 |  | 4 |
| *Arctium lappa* (burdock) | F | 1 | Y | 3 |
| *Brassica napus* (oilseed rape) | F | 1 | Y | 2 |
| *Capsella bursa-pastoris* (shepherd's purse) | F | 0 |  | 2 |
| *Centaurea nigra* (common knapweed) | F | 1 | Y | 5 |
| *Cerastium fontanum* (mouse ear chickweed) | F | 0 |  | 2 |
| *Cirsium vulgare* (spear thistle) | F | 1 | Y | 10 |
| *Crataegus monogyna* (common hawthorn) | F | 1 | Y | 4 |
| *Epilobium tetragonum* (square stem willowherb) | F | 1 |  | 6 |
| *Galeopsis tetrahit* (common hemp nettle) | F | 1 | Y | 4 |
| *Galium aparine* (cleavers) | F | 0 |  | 3 |
| *Geranium dissectum* (cut-leaved cranesbill) | F | 1 | Y | 5 |
| *Heracleum sphondylium* (common hogweed) | F | 1 | Y | 9 |
| *Hypericum perforatum* (St John's wort) | F | 1 |  | 5 |
| *Lamiastrum galeobdolon* (yellow archangel) | F | 1 |  | 1 |
| *Lamium purpureum* (red deadnettle) | F | 1 | Y | 9 |
| *Lonicera periclymenum* (common honeysuckle) | F | 1 | Y | 4 |
| *Matricaria recutita* (scented mayweed) | F | 1 |  | 1 |
| *Medicago lupulina* (black medic) | F | 1 |  | 2 |
| *Papaver rhoeas* (field poppy) | F | 1 |  | 4 |
| *Pentaglottis sempervirens* (green alkanet) | F | 1 |  | 1 |
| *Peucedanum palustre* (milk parsley) | F | 0 |  | 1 |
| *Primula veris* (cowslip) | F | 1 |  | 3 |
| *Prunus spinosa* (blackthorn) | F | 1 |  | 10 |
| *Ranunculus ficaria* (lesser celandine) | F | 1 |  | 1 |
| *Raphanus raphanistrum* (wild radish) | F | 1 | Y | 4 |
| *Senecio jacobaea* (ragwort) | F | 1 | Y | 8 |
| *Senecio vulgaris* (groundsel) | F | 1 |  | 5 |
| *Silene dioca* (red campion) | F | 1 |  | 1 |
| *Silene latifolia* (white campion ) | F | 1 |  | 3 |
| *Sinapis alba* (mustard) | F | 1 |  | 1 |
| *Sisymbrium officinale* (hedge mustard) | F | 0 |  | 2 |
| *Solanum dulcamara* (bittersweet) | F | 1 | Y | 5 |
| *Sonchus arvensis* (perennial sow thistle) | F | 1 |  | 6 |
| *Stachys palustris* (marsh woundwort) | F | 1 | Y | 3 |
| **Plant Species** | **Recorded in field and/or gardens** | **Likely usage by bees** | **Observed visitation by bees** | **No. sites where recorded** |
| *Stachys sylvatica* (hedge woundwort) | F | 1 | Y | 2 |
| *Stellaria graminea* (lesser stitchwort) | F | 0 |  | 3 |
| *Stellaria holostea* (greater stitchwort) | F | 0 |  | 8 |
| *Stellaria media* (common chickweed) | F | 0 |  | 1 |
| *Trifolium pratense* (red clover) | F | 1 | Y | 2 |
| *Tripleurosperumum inodorum* (mayweed) | F | 0 |  | 11 |
| *Viburnum lantana* (wayfaring tree) | F | 0 | Y | 3 |
| *Anthriscus sylvestris* (cow parsley) | F & G | 0 |  | 15 |
| *Cirsium arvense* (creeping thistle) | F & G | 1 | Y | 12 |
| *Convolvulus arvensis* (field bindweed) | F & G | 1 | Y | 13 |
| *Epilobium angustifolium* (rosebay willowherb) | F & G | 1 | Y | 10 |
| *Epilobium ciliatum* (american willowherb) | F & G | 1 |  | 2 |
| *Geranium pratense* (meadow cranesbill) | F & G | 1 | Y | 9 |
| *Glechoma hederacea* (ground ivy)^2^ | F & G | 1 | Y | 8 |
| *Hyacinthoides non-scripta* (bluebell) | F & G | 1 |  | 9 |
| *Lamium album* (white deadnettle) | F & G | 1 | Y | 10 |
| *Lapsana communis* (Nipplewort) | F & G | 0 |  | 11 |
| *Leontodon* spp (hawksbit) | F & G | 1 |  | 9 |
| *Leucanthemum vulgare* (oxeye daisy) | F & G | 1 |  | 3 |
| *Lotus corniculatus* (bird's foot trefoil) ^3^ | F & G | 1 | Y | 4 |
| *Myosotis* spp (forget me not) | F & G | 0 |  | 13 |
| *Passiflora pardifolia* (Passion Flower) | F & G | 1 | Y | 1 |
| *Prunus avium* (Wild Cherry) | F & G | 1 |  | 3 |
| *Ranunculus repens* (buttercup) | F & G | 0 |  | 12 |
| *Rosa canina* (dog rose) | F & G | 1 | Y | 10 |
| *Rubus fruticosus* (bramble) | F & G | 1 | Y | 16 |
| *Sambucus nigra* (elder) | F & G | 1 | Y | 8 |
| *Sinapis arvensis* (charlock) | F & G | 1 | Y | 1 |
| *Taraxacum officinale* (dandelion) | F & G | 1 | Y | 14 |
| *Trifolium dubium* (lesser trefoil) | F & G | 1 |  | 2 |
| *Trifolium repens* (white clover) | F & G | 1 | Y | 7 |
| *Veronica persica* (field speedwell) | F & G | 0 |  | 14 |
| *Viola arvensis* (field pansy) | F & G | 1 |  | 4 |
| *Abelia × grandiflora* (glossy Abelia) | G | 1 |  | 1 |
| *Acacia* spp (Acacia) | G | 1 |  | 1 |
| *Acanthus spinosus* (bear's breeches) | G | 1 |  | 1 |
| *Alchemilla* spp (lady's mantle) | G | 1 |  | 1 |
| *Anemone hupehensis* (windflower) | G | 1 |  | 2 |
| *Aquilegia* spp (columbine) | G | 1 |  | 2 |
| *Armeria* spp (sea pink plantain) | G | 1 |  | 1 |
| *Astilbe* spp (Astilbe) | G | 0 |  | 1 |
| *Aubretia* spp (Aubretia) | G | 0 |  | 3 |
| *Bellis perennis* (common daisy) | G | 0 |  | 3 |
| **Plant Species** | **Recorded in field and/or gardens** | **Likely usage by bees** | **Observed visitation by bees** | **No. sites where recorded** |
| *Berberis* spp (Berberis) | G | 1 | Y | 1 |
| *Borago officinalis* (borage) | G | 1 | Y | 1 |
| *Buddleja* spp (Buddlea) | G | 1 | Y | 2 |
| *Camelia* spp (Camelia) | G | 0 |  | 1 |
| *Campanula medium* (Canterbury bell) | G | 1 |  | 1 |
| *Campanula* spp (giant harebell) | G | 1 |  | 1 |
| *Ceanothus* spp (Ceanothus) | G | 1 |  | 1 |
| *Centaurea* cyanus (cornflower) | G | 1 |  | 1 |
| *Centaurea montana* (mountain cornflower) | G | 1 |  | 1 |
| *Cerasticum tomentusum* (snow-in-summer) | G | 0 |  | 1 |
| *Choisya ternata* (Choisya) | G | 1 | Y | 2 |
| *Chrysanthemum* spp (Chrysanthemum) | G | 0 |  | 2 |
| *Cistaceae* spp (rock rose) | G | 1 | Y | 2 |
| *Clematis montana* (anemone clematis) | G | 0 |  | 1 |
| *Common Vetch* (common vetch) | G | 1 |  | 1 |
| *Convallaria majalis* (lily of the valley) | G | 1 |  | 2 |
| *Cordyline* spp (Cordyline) | G | 0 |  | 1 |
| *Corydalis lutea* (yellow corydalis) | G | 1 |  | 1 |
| *Cotinus coggygria* (smoke bush) | G | 1 |  | 1 |
| *Cotoneaster × watereri* (Waterer's cotoneaster) | G | 1 |  | 2 |
| *Crocosmia* spp (montbretia) | G | 1 |  | 1 |
| *Daucus carota* (wild carrot) | G | 0 |  | 1 |
| *Dianthus* spp (pinks) | G | 1 |  | 2 |
| *Diascia* spp (twinspur) | G | 1 |  | 1 |
| *Epilobium montanum* (broad-leaved willowherb) | G | 1 |  | 1 |
| *Erica tetralix* (cross-leaved heather) | G | 1 |  | 1 |
| *Escallonia* spp (Escallonia) | G | 1 |  | 1 |
| *Fuchsia* spp (fushsia) | G | 1 |  | 2 |
| *Geranium platypetalum* (broad-petalled geranium) | G | 1 | Y | 4 |
| *Geranium robertianum* (herb robert) | G | 1 |  | 1 |
| *Geranium sanguineum* (bloody cranesbill) | G | 1 |  | 1 |
| *Geum urbanum* (wood avens) | G | 1 |  | 2 |
| *Helianthus annuus* (sunflower) | G | 1 | Y | 1 |
| *Helleborus orientalis* (hellebore) | G | 1 |  | 1 |
| *Heuchera* spp (firefly) | G | 0 |  | 1 |
| *Hydrangea macrophylla* (bigleafed hydrangea) | G | 0 |  | 1 |
| *Iris germanica* (german iris) | G | 0 |  | 1 |
| *Kerria japonica* (japanese yellow rose) | G | 0 |  | 1 |
| *Kolkwitzia amabilis* (beauty bush) | G | 1 |  | 2 |
| *Lamprocapnos spectabilis* (bleeding heart) | G | 0 |  | 1 |
| *Laurus nobilis* (bay laurel) | G | 0 |  | 2 |
| **Plant Species** | **Recorded in field and/or gardens** | **Likely usage by bees** | **Observed visitation by bees** | **No. sites where recorded** |
| *Lavandula* spp (lavender) | G | 1 | Y | 1 |
| *Lunaria rediviva* (perennial honesty) | G | 1 |  | 1 |
| *Lupinus* spp. (lupin) | G | 1 | Y | 1 |
| *Lysimachia* spp (yellow loosestrife) | G | 1 |  | 1 |
| *Lythrum* spp (purple loosestrife) | G | 1 |  | 1 |
| *Malus domestica* (apple) | G | 1 |  | 2 |
| *Medicago sativa* (alfalfa) | G | 1 |  | 1 |
| *Muscari* spp (grape hyacinth) | G | 1 |  | 2 |
| *Penstemon* spp (beardtongue) | G | 1 |  | 1 |
| *Persicaria* spp (pinkweed) | G | 0 |  | 1 |
| *Phlox* spp (Phlox) | G | 1 |  | 1 |
| *Potentilla fruticosa* (Potentilla) | G | 1 |  | 1 |
| *Primula vulgaris* (primrose) | G | 1 |  | 2 |
| *Prunella vulgaris* (self heal) | G | 1 |  | 1 |
| *Prunus Laurocerasus* (english laurel) | G | 1 | Y | 2 |
| *Pulmonaria* spp. (lungwort) | G | 1 |  | 1 |
| *Rhodedendron* spp. (Rhodedendron) | G | 1 |  | 1 |
| *Rosa* spp (ornamental garden rose) | G | 0 |  | 2 |
| *Rosmarinus officinalis* (rosemary) | G | 1 | Y | 3 |
| *Salvia officinalis* (common Salvia) | G | 1 |  | 2 |
| *Sanguisorba officinalis* (great burnet) | G | 1 |  | 1 |
| *Saxifraga x urbium* (London pride) | G | 0 |  | 3 |
| *Sisyrinchium bermudianum* (Bermuda blue-eyed grass) | G | 1 |  | 1 |
| *Solidago* spp. (goldenrod) | G | 1 |  | 1 |
| *Spirea* spp. | G | 0 |  | 2 |
| *Stachys densiflora* (common bettony) | G | 1 |  | 2 |
| *Tulipa* spp (tulip) | G | 0 |  | 2 |
| *Weigelia* spp. (Weigelia) | G | 1 | Y | 2 |
